# Twenty Years of Ferroportin Disease: A Review or An Update of Published Clinical, Biochemical, Molecular, and Functional Features

**DOI:** 10.3390/ph12030132

**Published:** 2019-09-09

**Authors:** L. Tom Vlasveld, Roel Janssen, Edouard Bardou-Jacquet, Hanka Venselaar, Houda Hamdi-Roze, Hal Drakesmith, Dorine W. Swinkels

**Affiliations:** 1 Department of Internal Medicine, Haaglanden MC-Bronovo, 2597AX The Hague, The Netherlands; 2 Department of Laboratory Medicine, Translational Metabolic Laboratory, Radboud University Medical Center, P.O. Box 9101, 6500 HB Nijmegen, The Netherlands; 3 Liver Diseases Department, French Reference Centre for Rare Iron Overload Diseases of Genetic Origin, University Hospital Pontchaillou, 35033 Rennes, France; 4 Centre for Molecular and Biomolecular Informatics, Radboud Institute for Molecular Life Sciences, Radboud, University Medical Center, P.O. Box 9191, 6500 HB Nijmegen, The Netherlands; 5 Molecular Genetics Department, French Reference Centre for Rare Iron Overload Diseases of Genetic Origin, University Hospital Pontchaillou, 35033 Rennes, France; 6 MRC Human Immunology Unit, Weatherall Institute of Molecular Medicine, University of Oxford, Oxford OX39DS, UK

**Keywords:** SLC40A1, ferroportin, iron overload, non-HFE, ferritin, hemochromatosis

## Abstract

Iron overloading disorders linked to mutations in ferroportin have diverse phenotypes in vivo, and the effects of mutations on ferroportin in vitro range from loss of function (LOF) to gain of function (GOF) with hepcidin resistance. We reviewed 359 patients with 60 ferroportin variants. Overall, macrophage iron overload and low/normal transferrin saturation (TSAT) segregated with mutations that caused LOF, while GOF mutations were linked to high TSAT and parenchymal iron accumulation. However, the pathogenicity of individual variants is difficult to establish due to the lack of sufficiently reported data, large inter-assay variability of functional studies, and the uncertainty associated with the performance of available in silico prediction models. Since the phenotypes of hepcidin-resistant GOF variants are indistinguishable from the other types of hereditary hemochromatosis (HH), these variants may be categorized as ferroportin-associated HH, while the entity ferroportin disease may be confined to patients with LOF variants. To further improve the management of ferroportin disease, we advocate for a global registry, with standardized clinical analysis and validation of the functional tests preferably performed in human-derived enterocytic and macrophagic cell lines. Moreover, studies are warranted to unravel the definite structure of ferroportin and the indispensable residues that are essential for functionality.

## 1. Introduction

Hereditary hemochromatosis (HH) type 4 or ferroportin disease (OMIM-code: 606069 Orphanet-code: 139491) is associated with variants in *SLC40A1* and inherited in an autosomal-dominant manner. Since 1999, ferroportin disease was classified into two entities [1,2,3], based on experimental in vitro models of genetic variants found in patients [4,5,6,7,8,9,10]. Classical ferroportin disease (Type 4A) is associated with loss-of-function (LOF) variants with diminished cell surface expression of ferroportin and lower iron export capacity, and is characterized by macrophage iron retention and iron restriction for erythropoiesis. It is clinically recognized by the presence of high serum ferritin concentrations with low to normal transferrin saturation (TSAT) and poor tolerance to phlebotomy. Non-classical (atypical, type 4B) ferroportin disease is associated with gain-of-function (GOF) variants that render ferroportin protein resistant to hepcidin, resulting in continued iron export, and leading to a phenotype that mimics classical HFE HH characterized by parenchymal (hepatocellular) iron overload with elevated serum ferritin and TSAT. 

Systematic and narrative reviews on ferroportin disease revealed the great variety in iron parameters among patients with ferroportin variants and emphasize the poor correlation between iron export capacity in functional studies and clinical characteristics (phenotype) [7,9,10,11] and question the pathogenicity of various variants [8,12].

To better guide the management of this disorder, a better understanding of the clinical and biochemical features of ferroportin disease and its underlying pathological mechanisms is required. In this report, we evaluated and describe clinical and laboratory data of patients reported between 1999 and June 2019, in whom the identified ferroportin variant was assumed to be associated with the defined state of iron overload. The relation between patient’s clinical features and the functional in vitro features of underlying variants is assessed in context of current knowledge on the function and structure of ferroportin and its regulatory mechanisms. We comment on the shortcomings of describing the phenotype based on case and family reports, and review the limited applicability of functional studies and available in silico prediction models to determine pathogenicity. We recommend the institution of a global registry with a (1) standardized diagnostic protocol, (2) validated functional tests, and (3) studies to establish the definite secondary and tertiary structure of ferroportin and residues involved in its binding with hepcidin for ubiquitination and to ferroportin for iron transport. 

## 2. Methods

### 2.1. Data Collection and Selection

#### Identification, Demographic Characteristics, and Iron Parameters 

A literature search was done using Medline and Embase (searching strategy: #1 (SLC40A1 or ferroportin or IREG or IREG-1 or FPN or FPN1 or non-HFE hemochromatosis), #2 (mutation or variant) from 1999 to June 2019. Papers with only an abstract in English were also selected. We included all the cases (including family studies) of ferroportin variants that are associated with unexplained elevated serum ferritin concentration. Since the aim of this analysis was to evaluate the relation between the clinical features and the functional in vitro characteristics in experimental cell lines of the various potentially pathogenic variants, we excluded variants for which these functional studies have not been performed, e.g., variants in promoter and untranslated regions, splicing variants as well as variants leading to synonymous amino acids such as Iso109Iso, Leu129Leu, and Val221Val [13,14,15,16,17]. In addition, we excluded patients with previously established neutral or (possibly) iron-modifying polymorphisms with a variant population frequency > 1%, such as the African Gln248His variant and variants in residues Leu348 and Leu384 [7,18,19,20,21].

Only patients with reported numerical value of at least transferrin saturation (TSAT) or ferritin concentration were selected. Demographic features, presenting symptoms, blood test results (hemoglobin (Hb), mean corpuscular volume (MCV), serum iron, TSAT, ferritin, and alanine aminotransferase (ALT)), hepatic iron content and hepatic histology, data on tolerability of phlebotomy (including the amount of iron removed), and the presence of p.Cys282Tyr and p.His63Asp HFE variants and other potential confounders, such as alcohol consumption and signs of metabolic syndrome, were collected. Organ iron accumulation as assessed by MRI and histologic examination was evaluated. Hepatic damage was histologically scored using the Meta-analysis of Histological Data in Viral Hepatitis (METAVIR) score (0 = no fibrosis, 1 = mild fibrosis (portal fibrosis without septa), 2 = moderate fibrosis (portal fibrosis and few septa), 3 = severe fibrosis (numerous septa without cirrhosis), and 4 = cirrhosis). Differences between categorical variables were compared with the Chi-square test with Yates correction while the comparison of continuous variables was done with the Mann-Whitney U-test and Student’s–t -test of independent means. A two-sided *p*-value < 0.05 was considered significant. The correlation between variables was determined by the Pearson correlation coefficient.

### 2.2. Variant Classification Based on Functional In Vitro Studies

Functional studies on iron export and/or ferroportin expression were performed in cell lines transfected with the ferroportin protein variant. We reviewed the reported functional studies on iron export capacity and ferroportin expression that were performed in cell lines transfected with the wild-type and the variant ferroportin protein. We assigned a variant as an LOF variant when the iron export capacity was significantly reduced, in comparison to the wild-type (WT) protein. The variant was assigned as a GOF variant when the iron transport capacity was preserved. When the addition of hepcidin significantly inhibited the iron export capacity and/or ferroportin expression, the variant was defined as hepcidin-sensitive, and the variant was hepcidin-resistant in the absence of a modulating effect of hepcidin. A variant was scored as “non-classified” when no relevant functional data were available, and as “conflicting” when data on the hepcidin effect were inconsistent or inconclusive.

### 2.3. Bioinformatics Prediction Software Analysis

To assess co-segregation in multiple affected family members, the simplified method of segregation analysis (SISA) was applied [22,23] and classified according to recent recommendations to define co-segregation to fit the guidelines of the American College of Medical Genetics and Genomics and the Association for Molecular Pathology (ACMG-AMP) [22,24]. Briefly, to determine the co-segregation we used the classification suggested by Jarvik et al. and the method to calculate the probability that the phenotype of a variant carrier is caused by chance rather than genotype, as suggested by Moller et al. [22,23]. This probability is described as (1/2)^n^, wherein n is the number of informative meiosis and will be the number of the carriers with the affected phenotype of interest and all the carriers in between them, minus one. The genome aggregation data base (gnomAD), was applied to determine of the allele frequency of a variant. In order to further determine the potential pathogenicity of the various variants three commonly used algorithms were applied [25]. Polyphen2 predicts the impact of amino acid substitution on the structure and function of human protein using physical and comparative considerations [26]. The method “Sorts intolerant from Tolerant” (SIFT) (http://www.blocks.fhcrc.org/sift/SIFT.html) classifies non-synonymous single nucleotide polymorphisms on the basis of the evolutionary conservation of amino acids within protein families [27]. Align-GVGD predicts variants in the query sequence based on a combination of Grantham Variation (GV) which measures the evolutionary variation at a particular position in the alignment and Grantham Deviation (GD), measuring the biochemical difference between the reference and amino acid encoded by the variant [28].

## 3. Evaluation and Analysis

### 3.1. Description of the Phenotype of Patients (Including Related Family Members) in Relation Findings in Functional Studies

#### 3.1.1. Patients Characteristics

##### Ferroportin Disease Occurs in Different Parts of the World and Has no Specific Symptoms 

We retrieved 359 individuals with 60 variants (59 missense variants and one deletion) in the ferroportin protein (Table 1). The reported patients, were predominantly male and of European descent. When reported, fatigue and arthralgia were the major presenting symptoms. Overall, patients had normal Hb and MCV with a serum iron level and TSAT at the upper range of normal and moderately elevated ferritin levels. 

Women had significantly lower Hb, TSAT, and serum ferritin concentration (Table 1). There was a weak correlation between age and TSAT (*r* = 0.1796) and serum ferritin (*r* = 0.3461) (Figure S1a–c). Probands were significantly older than affected family members (45.5 versus 34 years) with significantly higher (1896 versus 948 μg/L) serum ferritin levels (Table S1). Ten percent (20/191) of affected family’s members (6/14 males/females, median age 42 years) had no biochemical signs of iron overload (ferritin level ≤ 200 μg/L and normal TSAT). 

##### Modification by Additional Hereditary and Acquired Conditions

We found no effect of the presence of p.Cys282Tyr (13 heterozygotes) and p.His63Asp (35 heterozygotes, five homozygotes) HFE variants on iron parameters (Table S2). In only 84 patients, the current alcohol consumption was reported without demonstrable effect (Table S3). The reported clinical data were insufficient for the proper determination of the presence of metabolic syndrome to explore its modifying effect. 

### 3.2. Relation between In Vitro Functional Studies of Ferroportin Variants on Clinical Features 

We identified 27 different GOF variants (110 patients), 21 LOF variants (205 patients), and 12 unclassified variants (33 patients) (Table 2). A total of 355 cases involved heterozygosity for ferroportin variants. One homozygous Gly204Ser GOF variant was reported in a 52-year-old female with ferritin concentration of 5236 μg/L, TSAT of 100%, and symptoms of hepatic fibrosis revealed by radiology [15]. A patient with homozygosity for the GOF variant Arg561Gly had biochemical signs of severe iron overload reflected by a serum ferritin of 2750 μg/L and TSAT of 84% [29].

#### 3.2.1. Clear Distinct Phenotypical Features in Patients with GOF and LOF Variants 

**Figure 1:**
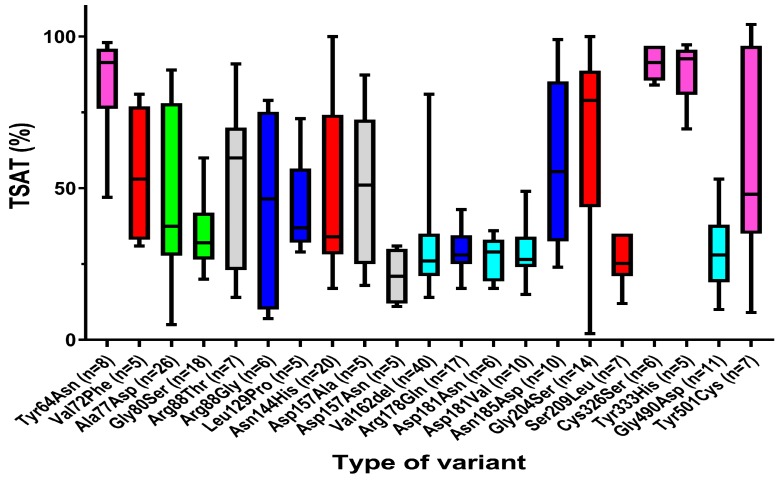
Box plots (Whiskers: min to max) of transferrin saturation (TSAT) in individuals with ferroportin variants. Only the variants with at least five reported TSAT levels in patients are included. Red: GOF without or conflicting data about hepcidin sensitivity; Pink: GOF hepcidin-resistant variants; Green: LOF without or conflicting data about hepcidin sensitivity; Light blue: LOF hepcidin-resistant variants; Blue: LOF hepcidin-sensitive variants; Gray: No data available.

Patients with GOF variants had significantly lower ferritin (755 μg/L versus 1595 μg/L) concentration (Table 3) and higher serum iron (36.0 μmol/L versus 17.5 μmol/L) concentration and TSAT (62% versus 32%) than patients with LOF variants. Inter-individual variation in TSAT levels was high in both LOF and GOF patients (Figure 1). In contrast to previous reports [7,105] and the prevailing opinion [10], patients with LOF and GOF variants were found to have a comparable Hb and prevalence of anemia (according to the World Health Organization criteria). While hepatic iron content was similar in patients with LOF and GOF variants, patients with LOF variants display iron deposition predominantly in macrophages (Kupffer cells), while the iron in GOF patients is predominantly present in hepatocytes, and is associated with more hepatic damage as reflected by the significantly higher serum ALT and amount of fibrosis (scored according to the METAVIR scale).

Long-term follow-up information and data to determine a difference in clinical course between GOF and LOF variant patients are limited [12,106]. We found three documented deaths related to ferroportin disease: fatal hepatocellular carcinoma in one GOF and one LOF variant patient [48,82]. One patient with a GOF mutation died of multiorgan failure due to widespread iron deposition [50].

#### 3.2.2. Strong Association of Hepcidin Sensitivity of Ferroportin Functional Gene Variants with Serum Iron Parameters

In vitro hepcidin sensitivity studies were performed in 45 variants. We identified seven hepcidin-resistant and seven hepcidin-sensitive GOF variants, and five hepcidin-resistant and four hepcidin-sensitive LOF variants (Table 2). Twelve LOF and 13 GOF variants were designated as uncertain/conflicting/unknown, including those variants with partial resistance or sensitivity.

**Figure 2:**
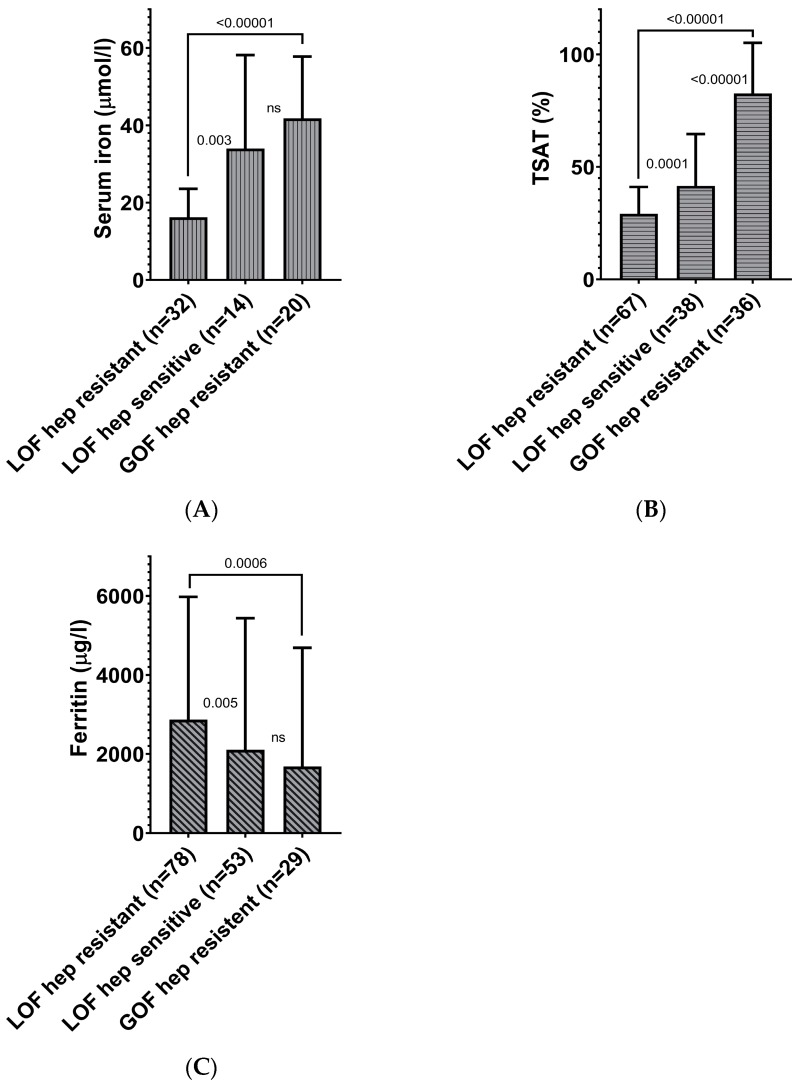
Iron parameters for the different ferroportin variants. Mean (+ SD) of (**A**) serum iron, (**B**) transferrin saturation, and (**C**) ferritin as measured in patient samples for the function of variant (LOF = loss-of-function, GOF = gain-of-function) and the effect of hepcidin (hep resistant = hepcidin-resistant, hep sensitive = hepcidin-sensitive) on the iron transport capacity, as assessed by in vitro functional tests. TSAT = transferrin saturation. The Student’s t-test was applied to determine the difference between serum iron and TSAT, while the Mann–Whitney U-test test was applied for ferritin. Displayed p values, serum iron, and TSAT are according to the Student’s t-test. Displayed p values ferritin according to the Mann–Whitney U-test.

Patients with hepcidin-resistant LOF variants had significantly lower TSAT (27% versus 35%) and serum iron (14.1 μmol/L versus 28.7 μmol/L) but higher ferritin (1810 μg/L versus 1066 μg/L) than patients with a hepcidin-sensitive LOF variant (Table S4, Figure 2). 

However, patients with hepcidin-resistant GOF variants had highly significant higher serum iron (44.0 μmol/L versus 14.0 μmol/L) and TSAT (92% versus 28%) and lower ferritin (642 μg/L versus 1810 μg/L) concentration than patients with hepcidin-resistant LOF variants (Table S5, Figure 2). All seven patients with a hepatocyte predominant iron deposition carried a hepcidin-resistant GOF variant, while all nine patients with a macrophage predominant iron deposition had a hepcidin-resistant LOF variant.

### 3.3. Therapeutic Considerations

In patients with primary iron overload, phlebotomy is the cornerstone of treatment. In 93 of 102 patients who were reported to be phlebotomized, the tolerance of the phlebotomy was reported. A total of 73 patients tolerated phlebotomy without side effects. In 17 of 20 patients with poor tolerance to phlebotomy, the cause of the intolerance could be analyzed. In 12 of the 14 patients with intolerance to phlebotomy due to the development of early anemia, phlebotomy could be continued by the application of a less intensive phlebotomy schedule. In one patient, anemia subsided despite the continuation of the same regimen. In three additional patients, the administration of erythropoietin resulted in an increase of Hb and continuation of the phlebotomy regimen [59,107]. In two patients, phlebotomy was discontinued because of the occurrence of anemia. Treatment with deferasirox resulted in a dramatic decrease in serum ferritin concentration in two patients, but was not successful in another [52,76,96]. 

In contrast to the current consensus [1,2,4,6,8,40,71,83,92,105], patients with LOF variants were not less tolerant to phlebotomy than patients with GOF variants (Table 3). Nevertheless, in five of 22 (23%) patients with hepcidin-resistant LOF variants the reported phlebotomy was poorly tolerated (Table S5) due to early anemia.

## 4. Critical Annotations on Pathogenicity of Variants

In Table 4, we depicted the potential pathogenicity of the various variants derived from available clinical, epidemiological, and genetic data from the original reports. We scored the likelihood of the pathogenicity of a variant based on the available data from the genome aggregation data base (gnomAD), data derived from the functional tests, at the molecular level in context of the current knowledge of the function and structure of the ferroportin molecule, and based on three in silico predicting models.

### 4.1. Shortcomings in Description of Clinical Phenotypes 

The collected data to describe the clinical phenotype were derived from extensively documented family studies to isolated patients with minimal reported clinical data as part of national surveys of patients with iron overload. So, the available clinical, biochemical, radiological, and histological data were the most heterogenous. In addition, in family studies, relatives were more than 10 years younger with a milder phenotype, including 11% with normal iron parameters. In many patients, a genetic diagnosis was made after referral, and reported iron parameters may potentially have been affected by previous phlebotomies. The demonstrated lack of effect on the presence of additional acquired iron-modulating conditions must be interpreted with caution in view of insufficient reported data. The presence of polymorphisms in ferroportin may affect the degree of iron overload in homozygotic patients for p.Cys282Tyr variants in the HFE molecule [13]. The lack of effect of presence of HFE variants on the iron parameters in our analysis is likely to be explained by the absence of patients with homozygotic p.Cys282Tyr HFE variants.

The phenotype of a number of reported variants raised doubt on the pathogenicity of these variants. An elevated TSAT is considered to be the hallmark of GOF variants. In 41 of 116 GOF patients, TSAT was normal. Since 13 of them also had a ferritin level ≤ 200 μg/L, 11% of GOF patients had normal serum iron parameters, especially patients with Asn144His (8/20 pts) and Val72Phe (2/5 pts) variants. Also, 7/9 reported patients with the seven hepcidin-sensitive GOF variants had a normal TSAT. The Gly204Ser GOF variant displayed evident pathologic iron parameters in a homozygous patient, with normal iron parameters in the two heterozygous relatives, and four of 14 additional reported patients had normal TSAT. Six of 197 LOF patients had normal reported ferritin level including 3/5 Leu129Pro variant patients. In a 10-year follow-up study of a proband and his father with a Arg489Ser variant, the serum iron spontaneously decreased with a slight reduction in the MRI estimated HIC [12].

### 4.2. Shortcomings in Establishment of Pathogenicity

#### 4.2.1. Co-Segregation with Disease in the Family

SISA was applicable for 26 variants and hampered by the small pedigrees in most reports. In 13 variants, there was no or only supporting evidence for pathogenicity; in nine variants, evidence was moderate or strong, and for four variants, the score for pathogenicity was inconclusive (Table 4). 

#### 4.2.2. Variant Occurrence and Allele Frequency

In the original reports, variant occurrence in healthy controls was determined for 37 variants in relatively small cohorts (range 40 to 734 persons) with an established occurrence of < 1/100 for 26 variants. In the genome aggregation data base (gnomAD, [25]), 213 missense variants were observed with subsequent determination of the allele frequency. Of these, only 13 variants displayed clinical symptoms to be reported and analyzed in the current review. Notably, 47/60 variants in our analysis are so rare that the allele frequency is not depicted in gnomAD. 

#### 4.2.3. Limitations of Functional In Vitro Tests

In most of the transfection studies HEK293T, a human embryonic kidney cell line, was used [6,8,11,31,32,33,35,39,40,41,42,46,55,56,57,83,87,93] and occasionally *Xenophus* oocytes, colon carcinoma CaCo-2, or hepatoma HuH7 cell lines [6,57,66]. Protocols are not standardized. Iron loading was done with 1 to 2 mg/mL of holotransferrin [8,41], 1 to 4 mg/mL of ^55^Fe [8,33,35,39,46], 20 to 40 μg/mL of ^59^Fe bound to transferrin [31,40,42], nitrilotriacetate [46] or ascorbate [66], or 10 to 100 μM of ferric ammonium citrate [6,32,55,57,83,93] for 24 h before to 48 h [8,31,32,33,39,40,42,46,55,66,83,87] after transfection. Cells were lysed 4 to 72 h after iron loading [8,31,33,46,56]. Iron export capacity was determined either by the measurement of radioactive Fe [8,31,33,35,39,40,42,46,56,66] or ferritin concentration [6,8,11,32,33,35,41,42,55,57,83,87,93] in lysed cells [6,32,33,35,40,42,57,93] or in the supernatant [8,11,31,33,35,39,46,56,83]. Results obtained in cells transfected with the variant ferroportin were compared with results in untransfected cells and in cells transfected with wild-type ferroportin. The effect of hepcidin on iron export capacity and the expression of the variant ferroportin protein were analyzed. Experimental conditions are also not standardized; specific differences include: (i) applied hepcidin concentration, which varied between 0.01 and 10 µM, and (ii) incubation times, which varied between 3 and 56 h [11,31,32,33,34,39,41,42,55,56,83,87,93]. The membrane expression of variant ferroportin proteins was determined by surface biotinylation and Western blotting [8,39], immunofluorescence [6,31,32,33,40,42,46,55,56,57,65,87] and confocal microscopy [11,34,41,83], or flow cytometry [31,35,41,42,56]. These differences may be responsible for the variable results reported on the effect of hepcidin on the membrane expression and iron export capacity of cells transfected with WT ferroportin.

In a significant number of the tested variants, the effect of hepcidin was determined by means of ferroportin expression and not by changes in iron export. Furthermore, in the vast majority of the transfected LOF variants, the membrane expression of the protein was strongly reduced or even absent, and therefore the effect of hepcidin is difficult to determine reliably. In a number of variants, the inhibitory effect of hepcidin was tested at various concentrations of hepcidin and/or exposure times, which resulted in a time and/or concentration dependent effect, ranging from hepcidin resistant to more or less hepcidin sensitive [6,30,42,65]. Therefore it is not unthinkable that the same variant is considered hepcidin resistant by one investigator and hepcidin sensitive by the other, depending on the design of the applied assay.

So, large inter-assay variation may not only lead to conflicting results in ferroportin variants tested in multiple studies, but also to reluctance to draw firm conclusions for data obtained in a single study. In addition, our definition of LOF (i.e. a significant reduced iron transport in functional tests) and hepcidin sensitivity (i.e. a significant reduction in iron transport or ferroportin expression in functional assays upon the addition of hepcidin) may lead to discrepancies in the interpretation of the functional characterization of some individual variants such as Gly204Arg and Arg296Gln, between us and the authors of the original publication [51].

#### 4.2.4. Lack of Concordance between Various In Silico Prediction Models

As illustrated in Table 4, the concordance between the three methods is poor, with a weak albeit significant correlation between SIFT and Align-GVGD (*r* = 0.5441), polyphen2 and SIFT (*r* = 0.3832), and polyphen2 and Align-GVGD (*r* = 0.3066).

### 4.3. Assessment of Pathogenicity of Ferroportin Variants on Molecular Level in Context of Current Knowledge on the Function and Structure of Ferroportin

#### 4.3.1. Ferroportin, the Protein, Its Variants, and Function: Current Knowledge

**Figure 3:**
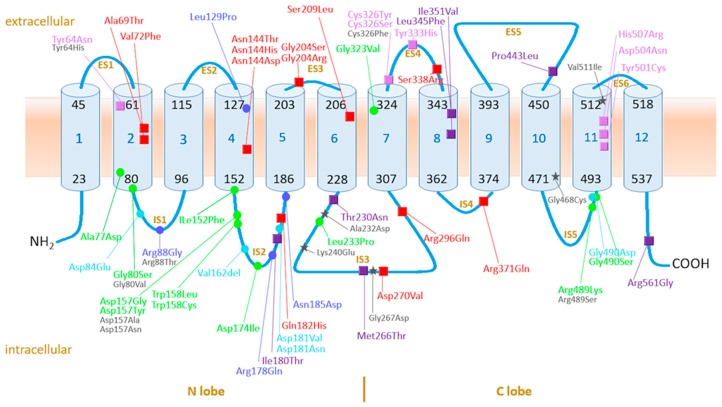
The two-dimensional (2D) structure of ferroportin protein adapted from Liu and Wallace [6,56], revealing 12 transmembrane helices and six extracellular and five intracellular segments. All the variants described in this review are shown as hepcidin-resistant (pink), hepcidin-sensitive or neutral (purple) and hepcidin conflicting/uncertain/unknown (red) GOF variants (squares). and hepcidin resistant (light blue), hepcidin sensitive (dark blue), and hepcidin conflicting-uncertain-unknown (green) LOF variants (dots), or non-classified variants (grey asterix).

Ferroportin is a 571 amino acid cation transporter of the major facilitator superfamily (MFS) encoded by the *SLC40A1* gene [46,108,109,110,111]. It is the only known cellular iron exporter, and primarily expressed in the basolateral membrane of duodenal enterocytes, macrophages, and hepatocytes [108,110,111,112]. In the most widely accepted secondary structure, ferroportin comprises 12 helices located in 12 transmembrane (TM) domains bound via six extracellular (ES) and five intracellular (IS) segments with a large intracellular loop between the sixth and seventh transmembrane helix and intracellularly located N and C terminus [6,8,34,46,56,113]. Although the amount of polymerization is still a matter of debate, most investigators assume that ferroportin is expressed on the membrane as a dimer [6,34,35,55,114]. Available three-dimensional models are based on comparison with ferroportin from other species with only 10-24% sequence homology and 40% similarity [8,39,46,115]. Recent studies reveal an open-inward and an open-outward structure with an intracellular and extracellular gate between the sixth and seventh transmembrane domain. Extrapolating the experimental model to the human protein reveals that residues Asp84, Arg88, Asp157, Asn174, Gln481, Glu486, and Arg489, which are located at IS1, IS2 and IS5, are important in intracellular gate interaction, while the residues Phe44, Val48, Val 51, Leu58, Asp325, Thr329, Leu342, and Phe520, which are located at TM1, TM12, ES1 and ES4, are involved in extracellular gate interaction (Figure 3) [46,115]. It is postulated that intracellular iron is transported outside the cell, and that extracellular hepcidin is transported inside the cell through this gate via a so called “rocker-switch” mechanism [46,115,116].

Since sites containing an Asp, Glu, His, or Tyr residue are reported be involved in iron binding, Asp39, Glu52, Tyr54, Asp181, Tyr318, Asp325, Tyr331, Tyr501, and Glu518 are potential iron binding sites and Arg88, As157, Asn174, and Arg489 are involved in the intracellular gate interaction [46,115]. Functional studies revealed cation binding at Ser35, Asp39, Asn212, and Ser215 and site mutagenesis studies suggest that Asp39 and Asp181 are essential iron binding sites [46,115]. The residues Arg88, Ile152, and Asn174 are thought to be directly involved in iron egress [39].

Ferroportin-mediated cellular iron export is systemically regulated by the hepatocyte-derived peptide hormone hepcidin. The way hepcidin binding inhibits iron efflux is not fully elucidated [117,118,119]. Functional studies reveal the internalization of ferroportin upon hepcidin binding with subsequent endosomal and lysosomal degradation resulting in the diminution of cellular iron export, while three-dimensional (3D) structural studies suggest that hepcidin binding disrupts the conformational transition into the intracellular gate with the subsequent inhibition of the access of cytoplasmatic iron to the substrate binding-sites [115,120,121]. Site mutagenesis studies and the exploration of various three dimensional models suggests that the Phe324, Cys326, Tyr333, Asp504, and His507 residues are important binding and docking sites for hepcidin [32,39,46,115]. Recent structural models indicate that these hepcidin binding residues are clustered in the extracellular gate in the open-outward structure. Residues found to be involved in internalization include Tyr64, Gly80, andAsn144, and the residues Lys229, Lys253, and Lys269 are present in the large intracellular loop, and seem to be involved in the degradation of ferroportin [39,114,122].

**Figure 4:**
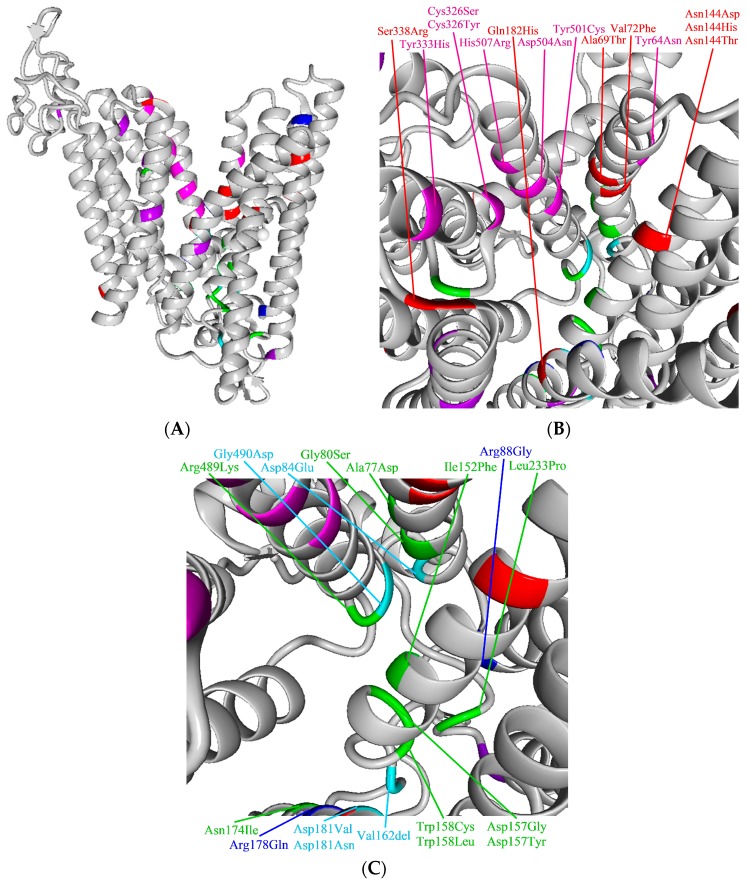
3D model of ferroportin. **(A)**. Overview. Ferroportin facing outward position. Top: extracellular; Bottom: intracellular. (**B)**. Localization of hepcidin-resistant GOF variants. View inside the “channel” from extracellular to intracellular. Six (Cys326Tyr, Cys326Ser, Tyr333His, Tyr501Cys, Asp504Asn, and His507Arg) of the seven residues of GOF hepcidin-resistant variants are located inside the channel. The Ala69, Val72, Asn144, Gln182, and Ser338 residues are also located inside the channel. However, these variants have conflicting or no data on hepcidin sensitivity. (**C)**. Clustering of LOF variants at the intracellular side of ferroportin. View inside the channel from extracellular to intracellular. Red: GOF without or conflicting data about hepcidin sensitivity; Pink: GOF hepcidin-resistant variants; Purple: GOF hepcidin-sensitive or neutral variants; Green: LOF without or conflicting data about hepcidin sensitivity; Light blue: LOF hepcidin-resistant variants; Blue: LOF hepcidin-sensitive variants. We used bacterial ferroportin for the 3D model (PDB file 5AYN).

In an effort to unravel the pathogenicity of ferroportin variants, we depicted the various variants in the structure of ferroportin molecule. For the two-dimensional (2D) model (Figure 3), we adapted the structure constructed by Liu and Wallace [6,56] and for three-dimensional (3D) model (Figure 4), we used the recently developed model (Bacterial ferroportin, PDB file 5AYN) and homology modeling in the Yet Another Scientific Artificial Reality Application (YASARA) [123] and WHAT IF Twinset [124] programs.

#### 4.3.2. GOF Variants

We identified Tyr64Asn, Cys326Tyr, Cys326Ser, Tyr501Cys, Asp504Asn, and His507Arg as definitely hepcidin-resistant GOF variants, and a co-culture of hepcidin with cells transfected with these variants does not lead to the internalization of the ferroportin variant with maintained iron export capacity and variant membrane expression [8,11,31,32,33,34,35,39,40,41,42,46]. Consequently, there is increased iron export from enterocytes and macrophages leading to hepatocellular iron overload. Observations in mice with the Cys326Ser variant reveal that increased dietary iron absorption is maintained by the upregulation of the Duodenal cytochrome b (Dcytb) and Divalent Metal Transporter 1 (DMT1) at the apical site of the enterocyte, which is likely to compensate for the pending intraenterocytic iron depletion [125]. For Cys326Tyr, Cys326Ser, Tyr501Cys and Asp504Asn, the blocked internalization of ferroportin on hepcidin exposure has been attributed to the impaired binding of hepcidin to ferroportin in the extracellular gate, while for Tyr64Asn and His507Arg—of which the location in relation to this gate varies between the available models—the impaired ubiquitination of hepcidin, which was most likely due to interference with conformation, is the most likely mechanism (Figure 4B) [30]. The Tyr333Ala, Tyr333His (both hepcidin-resistant variants in a functional test) and Tyr333Phe (hepcidin-sensitive) GOF variants display both membrane localization and intact iron export, but impaired ubiquitination after normal hepcidin binding; however, there are no data on configural changes due to presence of these variants [30,45].

The Ile180Thr, Thr230Asn, Met266Thr, Leu345Phe, Ile351Val, Pro443Leu, and Arg561Gly were designated as hepcidin-sensitive GOF variants. In functional studies, the iron export capacity, hepcidin uptake, and hepcidin-induced reduction of the cell surface expression of these variants were comparable with WT ferroportin [8,11,31,35]. None of these variants are located at residues that are known to be involved in hepcidin binding, ferroportin ubiquitination, or iron binding/egress. Notwithstanding that these seven variants behaved as WT ferroportin in functional studies and are located at residues without an established functional role, the reported 16 patients had otherwise unexplained mild iron overload, which was reflected by a median TSAT of 42.5% and serum ferritin concentration of 980 μg/l. Nevertheless, the pathogenicity of the variants may be questioned, and therefore, the variants may as well be classified as neutral [8].

#### 4.3.3. LOF Variants

Except for Leu129 and Gly323, the residues of the alterations that are associated with LOF variants are located at the intracellular site of the molecule, especially in the IS1, IS2, and IS5 (Figure 3) structures, which are involved in the formation of the intracellular gate responsible for iron transport (Figure 4C) [115]. In transfected cells, the majority of LOF variants are reported to be located intracellularly or have reduced membrane expression, although the data are not consistent for some variants (Table 2). Alteration in these residues may lead to conformational changes due to improper folding with mislocalization and disrupted intracellular gate formation. The substitution of Arg by Gly at residue 88 may lead to direct impaired iron handling with disrupted iron egress. That four hepcidin-sensitive variants display more or less intact membrane expression suggests intact extracellular gate formation enabling hepcidin binding. It was recently demonstrated that the hepcidin-sensitive LOF variant Arg178Gln, which is properly localized on the cellular membrane with disappearance after hepcidin exposure, is likely to form a non-covalent interaction between Arg178 and Asp473, which are located on the N and C lobe, respectively. This interaction may lead to a loss of stabilization of the open-outward conformation that is needed to preserve iron egress [84]. Residues of the four hepcidin-resistant LOF variants are not involved in hepcidin binding, so hepcidin resistance is likely to result from conformational changes in the extracellular gate, even though these variants have demonstrable but reduced membrane expression.

Overall, we conclude from that for most variants, clinical, functional, molecular, and in silico assessments of pathogenicity are not fully concordant, complicating the assessment of their likelihood of being the disease-causing variant (Table 4). For instance, the hepcidin-resistant GOF variants Tyr64Asn Cys326Ser, Cys326Tyr, Asp504Asn, and His507Arg are benign in two of the predicting in silico models, while clearly damaging variants in the three applied prediction models such as Leu129Pro and Ile180Thr were clinically likely to be benign. On the other hand, six out of the seven hepcidin-sensitive GOF variants had a low likelihood for pathogenicity both on clinical and molecular ground as well as in the predicting models.

### 4.4. Is the Mode of Iron Overload in LOF Variants Related to a Difference in Ferroportin Expression and Activation between Enterocytes and Macrophages?

Despite clear differences in iron distribution between patients with GOF and LOF variants, hepatic iron content and iron that is needed to be removed by phlebotomy to obtain a normalized iron phenotype are not found to differ, which might suggest that the total amount of absorbed iron in GOF and LOF patients is similar. Therefore, we hypothesize that in enterocytes, all the pathogenic ferroportin variants are associated with increased iron transport, irrespective of the (in vitro) function of the variant, while iron transport in macrophages and thereby the mode of systemic iron distribution is dependent on variant function. For patients with LOF variants, this hypothesis includes iron export being relatively decreased in macrophages, and being increased in enterocytes.

Ferroportin knock-out mice are not viable [110]. Mice with mono-allelic expression of wild-type (WT) ferroportin [111] and *TMPRSS6* knock-out mice with subsequent reduced ferroportin expression on enterocytic membrane, develop iron deficiency anemia with intra-enterocytic iron retention [126] and patients with *TMPRSS6* variants develop Iron Refractory Iron Deficiency Anemia (IRIDA) due to impaired intestinal iron absorption [127,128,129]. In contrast, patients heterozygous for LOF variants, which fail to export iron in functional tests, do not develop iron deficiency anemia; instead, they develop systemic, predominantly reticuloendothelial iron overload with limited but consistent proof for iron depletion in the enterocytes [79,88].

It has been recently suggested that differences in amount of intracellular iron turnover between enterocytes and macrophages may influence iron export capacity of these cells in patients with ferroportin disease due to LOF variants [130]. The monoallelic-expressed WT ferroportin protein would be sufficient to preserve iron export in cells with low iron turnover, such as enterocytes, but insufficient to maintain iron export capacity in cells with high iron turnover, such as splenic and hepatic macrophages, with subsequent intracellular iron retention in these cells. However, this hypothesis is insufficient to explain the state of iron overload. Functional consequences of a variant leading to the designation LOF is foremost established in HEK293T, which is a human embryonic kidney cell line, and are in accordance with events as observed in macrophages but fail to elucidate events that must be present in the enterocytes. So, HEK293T cells might not be the most appropriate experimental cell line to predict the functional consequences of at least LOF variants.

Experimental data provide evidence for differences in ferroportin expression and the activation between enterocytes and macrophages. Ferroportin is expressed on the enterocytic basolateral membrane and predominantly in intramacrophagic vesicles [108,131], and there are indications that the mode of hepcidin-induced internalization differs in macrophages [120,132]. 

Intracellular transcription and the translation of *SLC40A1* is primarily regulated by iron and oxygen in enterocytes and heme and iron in macrophages, [113,131,133]; also, the modulatory capacity by the iron-regulatory protein-iron-responsive element (IRP-IRE) system may differ between both cell types [134,135,136,137,138]. After iron egress from enterocytes, ferrous iron is oxidized by membrane-bound hephaestin, co-located with ferroportin, while ferrous iron release from macrophages is oxidized by circulating ceruloplasmin [139,140]. 

Ferroportin expression differs between enterocytes and macrophages in response to systemic regulatory stimuli such as erythropoietin and hepcidin, changes in iron state, and inflammation (hepcidin independent) in experimental animals, as well as in isolated cell cultures [132,141,142,143,144,145,146,147,148,149,150,151,152]. 

Taken together, these experimental data suggest a regulation difference of ferroportin production and activation, between enterocytes and macrophages [153]. However, the extrapolation of results obtained from experimental models to physiology must be done with caution.

## 5. Conclusions

We analyzed 60 low-frequency missense variants in the coding sequence of the ferroportin gene reported over the past 20 years, which has been assumed to be responsible for iron overload in individual patients reported in the English literature with or without subsequent evaluation of the relatives. The clinical and laboratory features of the patients (phenotype) with these ferroportin variants were correlated with data on variant properties in functional in vitro tests, which were available for 80% of the variants. However, the reported clinical features are often incomplete and functional tests are artificial, not standardized, or unvalidated, which impedes the interpretation of the correlation between the functional properties and phenotype. The establishment of variant pathogenicity is hampered by a lack of sufficiently reported data regarding the presence of iron-modifying confounders and because the outcomes of available in silico prediction models is limited in the absence of a fully elucidated structure and function. Therefore, it is likely that not all reported variants are indeed pathogenic, but from the available data, it is nearly impossible to accurately establish the pathogenicity of individual variants. Notwithstanding these limitations, we can conclude that ferroportin disease is a heterogeneous iron overload disorder. Clinical symptoms are non-specific, and mostly consist of fatigue and arthralgia. At presentation, patients are generally middle aged with strongly elevated ferritin concentrations and mildly elevated TSAT and iron concentrations. Iron parameters and the severity of iron overload show considerable inter-individual variation and are also related to gender, age, and type of variant. The mode of organ iron distribution is primarily determined by the effect of ferroportin variants on macrophage iron export, which is comparable with that observed in functional in vitro studies. LOF variants are associated with macrophage iron retention with a high serum ferritin and low to normal TSAT, in contrast to GOF variants, which are associated with high TSAT and hepatocellular iron deposition. These distinct phenotypes are typically present in patients with hepcidin-resistant variants. Patients with GOF variants had a higher prevalence and higher grade of hepatic fibrosis, indicating that parenchymal iron deposition is more toxic than macrophage iron overload. This corroborates the notion that high TSAT facilitates parenchymal iron deposition, which is considered to be more toxic than macrophage iron overload [154]. On the other hand, with regard to toxicity in macrophages, it has been stated that macrophage iron overload is resistant to iron withdrawal, and that iron-loaded Kupffer cells may contribute to the development of hepatic fibrosis and even carcinogenesis [106].

Since iron distribution patterns and clinical features of patients with hepcidin-resistant GOF variants are indistinguishable from patients with other types of HH, we support the proposition to categorize these variants as ferroportin-associated HH, and to confine the entity ferroportin disease to patients with LOF variants [116]. For both entities, repeated phlebotomies to normalize the iron parameters in order to prevent organ damage remain the mainstay of treatment. Although we found no increased intolerance to phlebotomy in patients with LOF variants, we advocate for the regular determination of Hb levels during the phlebotomy program, especially in patients with hepcidin- resistant LOF variants, to avoid the occurrence of early anemia.

The pathophysiology, especially of the LOF variants, is only partially understood. The iron handling within the various human cell types is not fully clarified, and the mechanisms by which ferroportin transports iron and is degraded are still not fully elucidated. To improve patient management, there is an unmet need for a better understanding of yet unresolved issues on the pathophysiology. A global registry, with a standardized diagnostic work-up and evaluation of the clinical, biochemical, radiological, and histological features of the liver and spleen, is needed, such as the registry sponsored by the European Association for the Study of the Liver (EASL) (http://non-hfe.com/) [155], a collaboration between various European Expert centers, or registries launched by the European Reference Network (ERN) of rare Hematological Disorders (EUROBLOODNET). Also, the histologic and molecular examination of enterocytes in duodenal biopsies of these patients by assessing iron content, as well as the expression of iron storage and transport proteins, will add to a better understanding of the iron handling. The secondary and tertiary structure of ferroportin as well as the identification of residues that are involved in hepcidin binding, ferroportin ubiquitination, and iron transport need to be further elucidated. Finally, the standardization of validated functional tests, which are preferably performed in human-derived enterocytic and macrophagic cell lines, will enable exploring iron transport and the modulating effect of hepcidin of known and yet unclassified variants, and will improve our insights of the pathophysiology of ferroportin disease.

## Figures and Tables

**Table 1:** Patient Characteristics. MCV: mean corpuscular volume, TSAT: transferrin saturation.

Characteristics	*n*	Value (Median)	Range
Patients	359		
Probands		68	
Relatives from probands		191	
Individual patients		88	
			
Gender	342		
Female		131	
Male		211	
			
Age (years)	322	41	2–87
			
Continent	352		
Europe		258	
North–South America		37	
Asia		44	
Australia/Oceania		13	
			
Presenting clinical symptoms	71		
Fatigue		34	
Elevated liver enzymes / hepatomegaly		30	
Joint complaints/arthralgia		36	
Miscellaneous		47	
			
Referral because of high ferritin level	131		
			
Hematological Parameters			
Hemoglobin (g/dL)^#^			
Female	60	13.1	7.5–16.3
Male	92	15.0	6.5–18.4
MCV (fl)			
Female	38	91	77–107
Male	46	91	70–108
Serum iron (µmol/L)			
Female	34	20.9	6.7–94.0
Male	56	26.3	6.2–86.0
TSAT (%)			
Female	116	31.5	2.0–100.0
Male	180	38.0	7.0–104.0
Ferritin (µg/L)			
Female	122	1026	4–8943
Male	201	1514	12–18695

Iron Parameters are Determined by Gender and Age. ^#^Significant difference between females and males (*p* < 0.05, also after correcting for multiple testing according to Bonferroni (<0.01).

**Table 2:** Functional and phenotypical characteristics of 359 patients with 60 ferroportin variants. GOF: gain of function, LOF: loss of function.

Protein	Iron Export		Hepcidin Effect on				TSAT		
Variant	Expression	Capacity	Expression	Export Capacity	Fate of Hepcidin	Patients (n)	Normal	High	Reference^%^
Tyr64Asn	PM	=	no	no	↓↓ubiquitination/uptake	8	-	8	[11,30,31,32,33,34,35,36]
Cys326Ser	PM	=	no	no	↓binding/no uptake	7	-	6	[30,32,34,37,38]
Cys326Tyr	PM	=	no	no	↓/no uptake	4	-	4	[8,31,34,35,39,40,41,42,43,44]
Tyr333His	PM	=	no	no		5	-	5	[45]
Tyr501Cys	PM	=	no	no	↓binding	7	2	5	[30,33,46]
Asp504Asn	PM	=	no	no	↓binding/uptake	2	-	2	[8,30]
His507Arg	PM	=	no	no	↓↓ubiquitination	4	-	4	[30,41,47]
Sum: 7	GOF		Hepcidin Resistant			37	2	34	
ILe180Thr	PM	=	yes	--	normal uptake	3	2	1	[8,48,49]
Thr230Asn	PM	=	yes	--	normal uptake	1	1	-	[8]
Met266Thr	PM	=	yes	--	normal uptake	1	1	-	[8]
Leu345Phe	PM	=	yes	--	normal uptake	1	1	-	[8]
Ile351Val	PM	=	yes	--	↑uptake	1	1	-	[8]
Pro443Leu	PM	=	yes	--	↑uptake	1	-	-	[8]
Arg561Gly	PM	=	yes	--	normal uptake	4	1	2	[8,29,50]
Sum: 7	GOF		Hepcidin Sensitive: Neutral			12	7	3	
Ala69Thr	PM	=	--	↓!	--	4	2	2	[42,51,52]
Val72Phe	PM	=	yes	↓!	↓binding	5	2	3	[30,53]
Asn144Asp	PM	=#	conflicting	no#	↓binding/uptake	2	-	1	[30,31,32,35,54]
Asn144His	PM	=	conflicting	yes	↓uptake	20	13	7	[3,6,31,34,35,39,40,42,55,56,57,58,59]
Asn144Thr	PM	=	conflicting	yes#	↓uptake	1	-	1	[6,31,32,34,60]
Gln182His	PM	=	conflicting	yes	normal uptake	2	1	-	[55,56,61]
Gly204Arg~	PM	=	no$	no$	--	1	-	1	[51]
Gly204Ser	PM	=	conflicting	no#	↓ubiquitination	14	4	10	[9,11,15,30]
Ser209Leu	PM	=	no$	no$	--	7	7	-	[62,63]
Asp270Val	PM	=#	yes	↓!	⇟ubiquitination	2	1	1	[14,30,64]
Arg296Gln~	PM	=	no$	⇟	--	1	1	-	[51]
Ser338Arg	PM	=	yes	↓!	⇟ubiquitination	1	-	1	[6,30,65]
Arg371Gln	IC/PM	=	no	yes	--	1	1	-	[11]
Sum: 13	GOF		Uncertain/Conflicting/Unknown			61	32	27	
Asp84Glu	--	↓	no	--	--	1	-	-	[43]
Val162del	IC/↓PM	↓	no	no	↓↓uptake	50	39	2	[6,8,35,43,51,55,56,66,67,68,69,70,71,72,73,74,75,76,77,78,79]
Asp181Asn	PM	↓	no	no	--	6	6	-	[51]
Asp181Val	↓PM	↓	no	no	--	10	10	-	[8,42,46,80]
Gly490Asp	IC/PM	↓	no	no	no uptake	12	9	3	[8,34,55,81]
Sum: 5	LOF		Hepcidin Resistant			79	64	5	
Arg88Gly	IC/PM	↓	yes	--	--	7	3	3	[8,11,82]
Leu129Pro	PM	↓	yes	⇟yes	--	5	3	2	[83]
Arg178Gln	PM	↓	yes	--	--	26	17	-	[75,82,84,85]
Asn185Asp	PM	↓	yes	--	--	19	4	6	[9,11,86]
Sum: 4	LOF		Hepcidin Sensitive			57	27	11	
Ala77Asp	IC/PM	↓	conflicting	not reliable@	no uptake	26	15	11	[1,2,8,11,34,35,39,41,42,56,57,66,71,73,87,88,89,90]
Gly80Ser	↓PM	↓^&^	not reliable@	not reliable@	normal uptake&	24	15	3	[8,57,73,87,88,91,92]
Ile152Phe	PM	↓	↓	↓	--	2	-	-	[39,93]
Asp157Gly	IC/PM	↓	conflicting	no&	--	4	3	1	[8,34,55,56,61]
Asp157Tyr	↓PM	↓	--	--	--	2	1	1	[8,94]
Trp158Cys	IC	↓	not reliable@	not reliable@	--	4	4	-	[41,62]
Trp158Leu	IC	↓	not reliable@	not reliable@	--	2	2	-	[11]
Asn174Ile	IC/PM	↓	conflicting	not reliable@	↓uptake	3	1	2	[34,39,46,87,88]
Leu233Pro	IC/↓PM	↓	not reliable@	--	--	3	1	2	[8,55,93]
Gly323Val	IC/PM	↓	conflicting	not reliable@	↓↓uptake	1	-	-	[34,55,56,61]
Arg489Lys	IC	↓	--	--	--	6	4	-	[40]
Gly490Ser	↓PM	↓	--	--	--	3	2	1	[8,35,82]
Sum: 12	LOF		Uncertain/Conflicting/Unknown			80	48	21	
Tyr64His						1	-	1	[95]
Gly80Val						2	2	-	[80]
Arg88Thr						7	3	4	[48]
Asp157Ala						5	2	3	[96,97,98]
Asp157Asn						5	5	-	[45,53]
Ala232Asp						2	2	-	[99]
Lys240Glu						1	-	1	[100]
Gly267Asp						1	1	-	[80]
Cys326Phe						1	-	1	[101]
Gly468Ser						3	3	-	[102]
Arg489Ser						4	2	2	[94,103]
Val511Ile						1	-	1	[45]
Sum: 12	Non-Classified					33	20	13	
								
Sum: 60						359	200	114	

%reference before the comma refers to reports on functional data; reference after the comma points to reports on clinical data; PM plasma membrane; IC intracellular; = comparable; -- no data available; ~ discrepancy in the classification with respect to the original report [51], ! dependent on the dose of hepcidin and time of exposure; # data not fully consistent; $ not strongly established; ^ In one study [104], this variant had diminished iron efflux; ↓↓ severe; ↓moderate/mild, ⇟ borderline impaired; @ interpretation is hampered since the variant is mainly localized intracellularly in the experiments; & data of de Domenico et al. [55,87] were disregarded in view of an unexpected discrepancy with other reports.

**Table 3:** Phenotypic features by ferroportin functional gene variant

Phenotypic Features	Gain-of-Function (*n* = 110)	Loss-of-Function (*n* = 216)	*p*
Age (years)	*n* = 93	*n* = 203	0.032^&^
median (range)	46 (2–80)	36 (6–87)	0.055^&&^
			
Gender (n)			
Female	35	82	
Male	66	126	ns
			
Hb (g/dL)	*n* = 39	*n* = 104	
median (range)	14.4 (9.5–16.5)	14.4 (10.1–18.4)	ns
			
Anemia			
Yes	4	14	
No	35	90	ns
			
MCV (fl)	*n* = 29	*n* = 50	
median (range)	93 (70–108)	91 (73–98)	ns
			
Serum iron (µmol/L)	*n* = 45	*n* = 51	0.0002^&^
median (range)	36.0 (8.0–74.0)	15.7 (6.2–94.0)	0.00017^&&^
			
TSAT (%)	*n* = 105	*n* = 174	< 0.0001^&^
median (range)	62 (2–104)	32 (5–99)	< 0.0001^&&^
			
Ferritin (µg/L)	*n* = 100	*n* = 208	< 0.0001^&^
median (range)	755 (4–15000)	1595 (24–21665)	0.0013^&&^
			
Iron Removed (g)	*n* = 10	*n* = 31	
median (range)	10.2 (2–24.4)	8.0 (1.6–80.0)	ns
			
Tolerance to Phlebotomy (n)			
Good	16	38	
Poor	2	13	ns
			
HIC(µg/g)	*n* = 19	*n* = 53	
median (range)	11718 (925–38665)	10052 (307–58590)	ns
Grade of fibrosis			
0–2	14	20	
3–4	10	2	0.012
			
Hepatic Iron distribution			
Hepatic (predominant)	18	7	< 0.00001^@^
Macrophagic (predominant)	0	28	
Mixed	10	16	< 0.00001^@@^
			
ALT (IU/L)	*n* = 31	*n* = 48	0.0026^&^
median (range)	52.0 (14.0–304.0)	29.5 (6.0–98.0)	0.004^&&^

HIC, hepatic iron content; ALT, alanine aminotransferase. ^&^Mann–Whitney U-test, ^&&^Student’s t-test of independent means, p is significant < 0.0033 when corrected for multiple testing according to Bonferroni. ^@^difference between hepatic and macrophagic, ^@@^difference between hepatic, macrophagic, and mixed.

**Table 4:** Likelihood of pathogenicity derived from the reported data, and current databases and prediction models.

							In silico Prediction		
Variant	Pheno-Type	Co-Segregation	VO/Controls	gnomAD	Functional	Molecular	Poly-phen2	SIFT	Align-GVGD
Tyr64Asn	**+++**	+++	0/100		**++++**	++	++	0	0
*Tyr64His*	*+++* ^&^					++	++	0	0
Ala69Thr	+++^&^	0			++	0	++	+	0
Val72Phe	**+**	+			+++	0	++	0	0
Ala77Asp	**+++**	**++,+++#**	0/100		**+++**	0	++	+	0
Gly80Ser	**++**		0/734		**+++**	+	++	+	0
*Gly80Val*	++		0/100			+	++	+	0
Asp84Glu	+++^&^				++	+	++	+	0
Arg88Gly	**+++**		0/734		++	+	++	+	0
*Arg88Thr*	**++**	+++	0/60			+	++	+	0
Leu129Pro	**0**	+			++++	0	++	+	+++
Asn144Asp	+++				**+++**	+	++	0	0
Asn144His	**0**	+++	0/200		**+++**	+	+	0	0
Asn144Thr	0^&^		0/100		**+++**	+	++	0	0
Ile152Phe	+++	+			+++	+	++	0	0
*Asp157Ala*	++	++				+	++	+	0
*Asp157Asn*	++	0				+	++	0	0
Asp157Gly	+++		0/80, 0/734		+++	+	+	0	0
Asp157Tyr	+++		0/734		+	+	++	+	+
Trp158Cys	+++	**0,** **+**	0/50		+++	0	++	+	++
Trp158Leu	+++				+++	0	+	0	0
Val162del	**++**	**+,+++**	0/100, 0/103, 0/734		**++++**	0	@	@	@
Asn174Ile	+++				**+++**	+	++	+	+++
Arg178Gln	**+++**	**0-+++** ^##^			++	+++	++	0	0
Ile180Thr	0	0	0/100	7.076^−5^	++	0	++	+	+++
Asp181Asn	**++**				**++**	+	++	+	+
Asp181Val	**++**	**+,++**	0/100, 0/734		**++**	+	++	+	+++
Gln182His	+++		0/80		+++	0	++	+	+
Asn185Asp	**+++**	**+,+++**	0/50		+	0	++	+	+
Gly204Arg	+++^&^				++	0	++	+	+++
Gly204Ser	**+**	+++	2/100	7.955^−6^	+++	0	++	0	0
Ser209Leu	**0**	++		1.485^−4^	+++	0	0	0	+
Thr230Asn	0^&^		0/734	1.096^−4^	++	0	+	0	0
*Ala232Asp*	0			4.95^−5^		0	+	0	+
Leu233Pro	+++	0	0/734		++	0	++	+	+++
*Lys240Glu*	+++^&^		0/50			0	0	+	0
Met266Thr	0^&^		0/734		++	0	0	+	0
*Gly267Asp*	++^&^		0/100	4.382^−5^		0	+	0	0
Asp270Val	+		4/100, 1/516	7.09^−5^	+++	0	0	0	0
Arg296Gln	0^&^				++	0	+	0	++
Gly323Val	+++^&^		0/80		**+++**	0	++	+	+++
*Cys326Phe*	+++^&^	0				++	++	0	+
Cys326Ser	**+++**	+++			**++++**	+++	+	0	0
Cys326Tyr	+++		0/800		**++++**	++	+	0	0
Tyr333His	**+++**		0/40		++	+	++	+	+++
Ser338Arg	+++^&^				**+++**	0	0	0	0
Leu345Phe	0^&^		0/734		++	0	++	0	0
Ile351Val	0^&^		0/734	2.125^−5^	++	0	0	0	0
Arg371Gln	0^&^			2.388^−5^	+++	0	0	0	0
Pro443Leu	0^&^		0/734	5.87^−4^	++	0	0	0	0
*Gly468Ser*	++	0		4.005^−6^		0	++	0	+
Arg489Lys	**+++**	+++	0/50		+	+	++	+	+++
*Arg489Ser*	+		0/734			+	++	+	+++
Gly490Asp	**+++**	0	0/734		**++++**	0	++	0	0
Gly490Ser	+++		0/734		+	0	++	0	0
Tyr501Cys	**+**		0/200	3.184^−5^	**++++**	+++	+	0	+
Asp504Asn	+++		0/734		++++	+++	++	0	0
His507Arg	+++	**+**	0/50		++++	++	++	0	0
*Val511Ile*	+++^&^					0	++	+	++
Arg561Gly	+		0/734	1.638^−3^	++	0	0	0	0

Open space: no data available. **Phenotype:** bold: at least five patients; 0 = < 50%, + = 51–80%, ++ = 81–99%, +++ =100% presence of an elevated TSAT in GOF patients and elevated ferritin in LOF patients. Variants in italic are unclassified; + = elevated TSAT and/or ferritin (but not in all patients), ++ = elevated TSAT or ferritin in all patients, +++ = elevated TSAT and ferritin in all patients. & only one reported patient. # enumeration of a limited number of studies; ## range in multiple studies. **Co-segregation** from pedigrees in the original reports using the simplified method of segregation analysis (SISA) according to the following scoring system: single family: 0 = no evidence, + = ≤1/8 (supporting), ++ = ≤ 1/16 (moderate), +++ = ≤ 1/32 (strong); bold: multiple family studies: 0 = no evidence, + = ≤ 1/4 (supporting), ++ = ≤ 1/8 (moderate), +++ = ≤ 1/16 (strong); all according to the recommendations of Jarvik et al. [22] to define co-segregation as criteria to fit the ACMG-AMP guidelines [24]. **VO/Controls:** Variant Occurrence in controls reported in the original reports. **GnomAD:** allele frequency derived from GnomAD data base [25]; the variants for which no allele frequency are given are absent in the database and thus extremely rare. **Functional:** + = effect of hepcidin not performed, ++ = effect of hepcidin established only on membrane expression or iron transport, +++ = conflicting or unreliable results on the effect of hepcidin, with reported fate of hepcidin, ++++ = established effect of hepcidin on membrane expression and iron export with/without reported fate of hepcidin; Bold = ≥ 3 independent studies. **Molecular:** 0 = variant present on a site without established role in ferroportin molecule, + = variant present at a site potentially involved in the formation of the intracellular and extracellular gate, internalization and degradation of the ferroportin molecule or at a site potentially involved in iron binding or egress, ++ = variant present at a site with established function in hepcidin binding, +++ = variant present at a site leading to established conformational changes in the structure of the ferroportin molecule as predicted in the available three-dimensional (3D) models. **In silico prediction:** Polyphen 2: 0 = benign, + = possibly damaging, ++ = probably damaging; “Sorts intolerant from Intolerant” (SIFT): 0 = tolerated, + = deleterious; Align-GVGD: 0 = class 0, + = Class 15, ++ = Class 25 and 35, +++ = Class 55 and 65; @ = It is not possible to test deletions in Polyphen2, SIFT, and Align-GVGD.

## Supplementary Materials

The following are available online at https://www.mdpi.com/1424-8247/12/3/132/s1, Figure 1S: Relation between age and serum iron/TSAT/ferritin, Table 1S: Characteristics and iron parameters of probands versus probands’ relatives, Table 2S: Phenotypical features by the reported presence of hereditary HFE and non-HFE iron overload conditions, Table 3S: Phenotypical features by the reported current use of alcohol, Table 4S: Phenotypical features of patients with functional LOF ferroportin variant by hepcidin sensitivity, Table 5S: Phenotypical features by hepcidin resistant GOF and LOF ferroportin variants.
