# Supplementary material for: Twenty Years of Ferroportin Disease: A Review or An Update of Published Clinical, Biochemical, Molecular, and Functional Features
Source: Pharmaceuticals (Basel). 2019 Sep 9;12(3):132. doi: 10.3390/ph12030132 (PMC6789780; doi:10.3390/ph12030132)
Supplement: Supplementary file 1 [file pharmaceuticals-12-00132-s001.pdf]

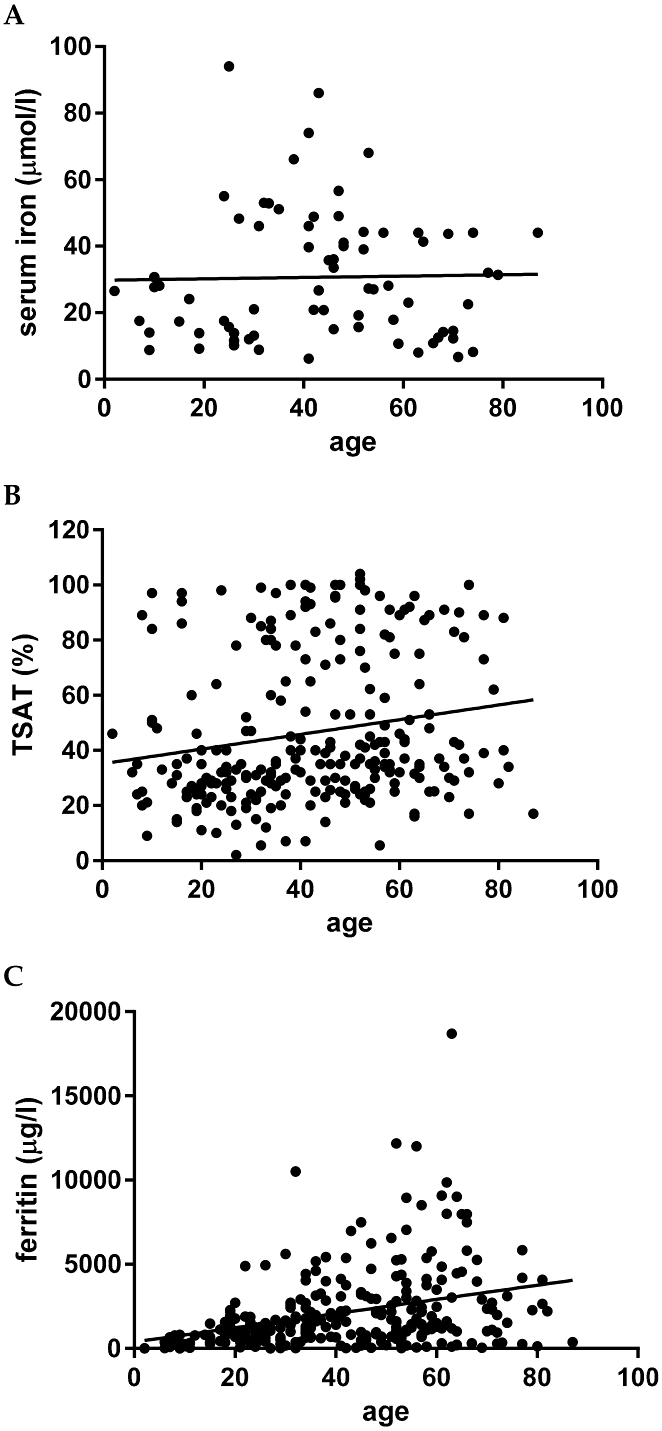

Figure 1S. Relation between age and (**A**) serum iron (r = 0.021, p = ns), (**B**) TSAT (r = 0.058, p = 0.003) and (**C**) serum ferritin (r = 0.327, p < 0.0001) in patients with reported ferroportin variants.

Table 1S. Phenotypical features by gender.

	**Female (n=123)**	**Male (n=195)**	***p***
**Age (years)**	n=112	n=184	
**median (range)**	36 (2-80)	44 (6-87)	ns
**Hb (g/dl)**	n=52	n=79	
**median (range)**	13.1 (7.5-15.6)	15.0 (9.5-18.4)	0.01
**MCV (fl)**	n=30	n=22	
**median (range)**	91 (77-107)	91 (73-108)	ns
**Serum iron (μmol/l)**	n=28	n=44	
**median (range)**	20.9 (6.7-94.0)	29.4 (6.2-86.0)	ns
**TSAT (%)**	n=108	n=165	
**median (range)**	31 (2-100)	40 (7-104)	0.0003
**Ferritin (μg/l)**	n=114	n=186	
**median (range)**	1011 (4-8943)	1452 (12-18695)	0.004
**Iron Removed (g)**	n=11	n=36	
**median (range)**	6.0 (3.2-24.4)	10.5 (1.6-80.0)	ns
**Tolerance to Phlebotomy**			
**Good**	17	41	
**Poor**	7	10	ns
**ALT (IU/L)**	n=26	n=27	
**median (range)**	22 (11-539)	52 (11-219)	ns
**HIC (μg/g dry weight)**	n=23	n=49	
**median (range)**	9542 (1083-36270)	10881 (307-58590)	ns
**Fibrosis (n)**	8	42	
**No fibrosis (n)**	11	24	ns
**Alcohol consumption**			
**Yes (n)**	7	21	
**No (n)**	17	38	ns

HIC: Hepatic Iron Content

Table 2S. Characteristics and iron parameters of probands versus probands' relatives.

	**Probands (n=66)**	**Relatives of probands (n=186)**	***p***
**Age (years)**	n=64	n=168	
**median (range)**	45 (9-71)	34 (2-87)	0.01
**Gender (n)**			
**female**	25	76	
**male**	40	109	0.007
**Hb (g/dl)**	n=34	n=60	
**median (range)**	14.3 (7.5-16.6)	14.1 (11.1-18.4)	ns
**Serum iron (μmol/l)**	n=19	n=41	
**median (range)**	27.0 (8-94)	26.5 (8.2-74)	ns
**TSAT (%)**	n=59	n=156	
**median (range)**	36 (5-100)	35 (2-104)	ns
**Ferritin (μg/l)**	n=63	n=173	
**median (range)**	1896 (65.1-12000)	932 (4-10510)	0.00002

HIC: Hepatic Iron Content

Table 3S. Phenotypical features by the reported presence of hereditary HFE and non-HFE iron overload conditions.

	Abnormalities (n=53)	No abnormalities (n=113)	*p*
**Age (years)**	n=45	n=110	
**median (range)**	32 (10-87)	41 (2-82)	ns
**Gender**			
**Female**	21	38	
**Male**	24	74	ns
**Hb (g/dl)**	n=20	n=52	
**median (range)**	14 (10.1-16.1)	14.8 (7.5-16.7)	ns
**Serum iron (µmol/l)**	n=26	n=43	
**median (range)**	27.4 (6.7-94)	32 (6.2-86)	ns
**TSAT (%)**	n=47	n=103	
**median (range)**	40 (2-99)	43 (5.6-104)	ns
**Ferritin (µg/l)**	n=53	n=100	
**median (range)**	1150 (4-14829)	1597 (12-12000)	ns
**HIC (µg/g dry weight)**	n=19	n=31	
**median (range)**	10044 (1083-45477)	13950 (999-58590)	ns

HIC: Hepatic Iron Content

Table 4S. Phenotypical features by the reported current use of alcohol.

	**Yes (n=28)**	**No (n=56)**	***p***
**Age (years)**	n=28	n=55	
**median (range)**	40.5 (19-72)	43 (6-79)	ns
**Gender**			
**Female**	7	17	
**Male**	21	38	ns
**Hb (g/dl)**	n=24	n=42	
**median (range)**	14,9 (11-16.6)	14.1 (7.5-18.4)	ns
**Serum iron (μmol/l)**	n=3	n=15	
**median (range)**	48.9 (19,2-53)	33.5 (10.7-86)	ns
**TSAT (%)**	n=28	n=48	
**median (range)**	35 (10-100)	40.5 (14-100)	ns
**Ferritin (μg/l)**	n=28	n=56	
**median (range)**	2101 (71-10510)	1586 142-12178)	ns
**HIC (μg/g dry weight)**	n=6	n=32	
**median (range)**	14816 (8750-21929)	10331 (3350-58590)	ns

HIC: Hepatic Iron Content

Table 5S. Phenotypical features of patients with functional GOF ferroportin variants by hepcidin sensitivity.

	GOF hepcidin resistant (n=32)	GOF hepcidin sensitive (n=10)	p
**Age (years)**	n=30	n=9	
**median (range)**	39.3 (2-77)	57 (37-72)	0.007
**Gender (n)**			
**Female**	14	2	
**Male**	18	8	ns
**Serum Iron (μmol/l)**	n=20	n=2	
**median (range)**	44.0 (8.8-74)	38.5 (28.1-49)	ns
**TSAT (%)**	n=31	n=9	
**median (range)**	92 (9-104)	42 (30-100)	0.0004
**Ferritin (μg/l)**	n=24	n=10	
**median (range)**	540 (12-3751)	980 (585-2960)	ns
**Response to phlebotomy (n)**			
**Good**	10	1	
**Poor**	0	1	ns

Table 6S. Phenotypical features of patients with functional LOF ferroportin variant by hepcidin sensitivity.

	LOF hepcidin resistant (n=68)	LOF hepcidin sensitive (n=57)	*p*
**Age (years)**	n=67	n=53	
**median (range)**	32 (6-74)	38 (6-87)	ns
**Gender (n)**			
**Female**	31	19	
**Male**	35	34	ns
**Serum Iron (µmol/l)**	n=21	n=14	
**median (range)**	14.0 (8.2-46)	28.7 (6.2-94)	0.01
**TSAT (%)**	n=57	n=38	
**median (range)**	28 (10-81)	35 (7-99)	0.0005
**Ferritin (µg/l)**	n=68	n=53	
**median (range)**	1853 (24-18695)	1066 (24-21665)	ns
**Response to phlebotomy (n)**			
**Good**	17	2	
**Poor**	8	4	ns

Table 7S. Phenotypical features by hepcidin sensitive GOF and LOF ferroportin variants.

	**GOF hepcidin sensitive (n=10)**	**LOF hepcidin sensitive (n=57)**	***p***
**Age (years)**	n=9	n=53	
**median (range)**	57 (37-72)	38 (6-87)	0.02
**Gender (n)**			
**Female**	2	19	
**Male**	8	34	ns
**Serum Iron (μmol/l)**	n=2	n=14	
**median (range)**	38.5 (28.1-49.0)	28.7 (6.2-94)	ns
**TSAT (%)**	n=9	n=38	
**median (range)**	42 (30-100)	35 (7-99)	ns
**Ferritin (μg/l)**	n=10	n=53	
**median (range)**	980 (585-2960)	1066 (24-21665)	ns
**Response to phlebotomy (n)**			
**Good**	1	2	
**Poor**	1	4	ns

Table 8S. Phenotypical features by hepcidin resistant GOF and LOF ferroportin variants.

	**GOF hepcidin resistant (n=32)**	**LOF hepcidin resistant (n=68)**	***p***
**Age (years)**	n=30	n=67	
**median (range)**	39.5 (2-77)	32 (6-74)	ns
**Gender (n)**			
**Female**	14	31	
**Male**	18	35	ns
**Serum Iron (μmol/l)**	n=20	n=21	
**median (range)**	44.0 (8.8-74.0)	14 (8.2-46.0)	0.00002
**TSAT (%)**	n=31	n=57	
**median (range)**	92 (9-104)	28 (10-81)	<0.00001
**Ferritin (μg/l)**	n=24	n=68	
**median (range)**	540 (12-3751)	1853 (24-18695)	0.003
**Iron distribution**			
**Macrophage**	0	9	
**Hepatocyte**	5	0	0.0005
**Response to phlebotomy (n)**			
**Good**	10	17	
**Poor**	0	5	ns
